# Enhancing Patient Safety Culture in Hospitals

**DOI:** 10.7759/cureus.51159

**Published:** 2023-12-27

**Authors:** Isha U Mistri, Ankit Badge, Shivani Shahu

**Affiliations:** 1 Hospital Administration, School of Allied Health Sciences, Datta Meghe Institute of Higher Education and Research (Deemed to Be University), Nagpur, IND; 2 Microbiology, Datta Meghe Medical College, Datta Meghe Institute of Higher Education and Research (Deemed to Be University), Nagpur, IND

**Keywords:** infection prevention, healthcare education healthcare quality, safety culture, patient safety climate, quality improvement

## Abstract

Patient safety has become a top priority for healthcare organizations. A better patient safety environment is associated with a lower probability of significant complications. Training programmers is critical to promoting patient safety and minimizing misunderstandings. The quality, performance, and productivity of the healthcare industry can be dramatically improved by changing the patient safety atmosphere operating within the hospital sector. Hospitals can significantly reduce medical errors and adverse events by implementing the program and training programmers to prioritize patient safety. This will improve patient outcomes and increase efficiency and effectiveness. Creating a patient safety culture within hospitals will contribute to a higher standard of care and improved overall performance in the healthcare industry. Hospitals can identify systemic problems and implement proactive measures to prevent future incidents by creating an environment in which healthcare professionals feel comfortable reporting errors. A patient safety culture encourages collaboration and open communication among healthcare teams leading to more effective and coordinated care.

## Introduction and background

Studies on patient safety have become more prevalent, coming from many different theoretical angles. Treatment and care are usually provided directly by healthcare professionals in patient settings. However, methods that include prescribed medications are commonly used to administer their medication in ambulatory settings, including primary care [1]. The patient safety environment is vital for better quality and patient-nurse safety, together with their scope of performance [2]. To protect patient safety, nurses must advocate patient safety, maintain patient care, and report unfavorable situations. Quality and safety issues persist and hamper the healthcare industry [3]. Patient safety is the goal of preventing medical errors and their adverse impacts on patients during the course of medication [4]. Unsafe interventions in medicine can cause harm, impairment, or even death of the patient [5]. The frequent occurrence of these events makes it clear that a patient safety culture must be maintained in the medical industry [6]. Identifying beliefs, attitudes, norms, and values in conjunction with their thresholds is important in establishing the highest degree of safety culture in the field of healthcare care [7]. Establishing the most stringent safety culture in the field of healthcare care requires knowing ideas, attitudes, norms, and values in combination with their thresholds [8]. Patient safety education is a rapidly growing topic because patient safety frameworks and curricula have just been constructed and implemented [9].

Without the concourse of front-line medical workers, interventions aimed at increasing patient safety are tricky and difficult to implement. Patient safety serves as a substitute for these interventions [10,11]. A patient safety environment includes complex interventions that involve the need for variations in individual work routines and healing processes as well as behavioral changes to be made on the part of the team or the individual for maximum acceptance from others. Most patients admitted to the facilities are complex, forcing multidisciplinary teams of professionals to treat them, with a combination of managerial employees and cutting-edge technologies. Consequently, offers to improve the availability of healthcare techniques require intense collaboration as well as cooperation between and among teams [12]. In hospitals and healthcare organizations, the prevention of infections has been observed in the quality control division. The healthcare epidemiologist frequently collaborates with the business executive who assesses quality and safety [13]. For healthcare improvement, healthcare institutions have also made concerted efforts to increase awareness of health quality and standard of care, and it offers the possibility of training in patient safety and quality [14]. The patient safety culture can be defined as the general attitudes and behavior habits related to the security functions in various organizational structures [15]. Patient safety culture can also represent the invisible and intangible underlying advantages because it can be analyzed and indicates the priority given to safety at different care healthcare levels [16].

Measuring the patient safety climate has blossomed into a technique for appreciating care processes and enhancing healthcare quality in particular [17]. A questionnaire often serves as the measuring device adopted for such measurements and 127 techniques were discovered in previous evaluations of patient safety culture measurement [18]. There were 11 key dimensions of patient safety identified by the review; however, no one methodology can capture all of them. The dimensions of leadership, perception of safety, teamwork and collaboration, safety systems, valuing safety, and resources and limits are the ones that arise most frequently from these assessment results [19]. This study aims to pinpoint and evaluate key features of the patient safety culture and associated factors among public health professionals.

## Review

Methods

To conduct a comprehensive literature search, we use the following databases: PubMed and Google Scholar. For this review, we use the following search terms: ("hospital safety," "healthcare quality," and "safety improvement." "Patient safety culture" OR "hospital safety") AND "improvement" OR "enhancement"). Articles published focusing on the enhancing patient safety culture on improving patient safety management, enhancing healthcare qualities, and ensuring patient safety were included in the current review a total of 47 articles were included in the study. The article types include systematic reviews, cross-sectional studies, qualitative studies, and descriptive studies.

Effective measures of patient safety climate

The most effective approach to envision the promotion of patient safety culture is as a constellation of interventions established in leadership, teamwork, and behavior modification compared to a specific technique, team, or technology [20]. One therapy, multiple interventions incorporated into a comprehensive approach, or a series of interventions can be used to promote a culture of patient safety. Several attempts to improve patient safety and treatment quality in acute care settings include the development of a culture of safety as an essential aspect [21].

A safety climate can be regarded as an external representation of a fundamental safety culture. Assesses how the workforce perceives the rules and procedures that suggest that safety has priority over other organizational objectives at their workplaces. The safety environment in the industry is measured by employing several measures. The accumulated statistics give leadership a different perspective on the condition of these systems for safety management and have applications for benchmarking and trend analysis. It has been suggested that the commitment of management to safety is the most relevant factor. Safety environments are of utmost importance for establishing a safe working environment for healthcare workers and for minimizing the risk of injury to patients. Evaluating the protection of healthcare personnel is the first step in establishing a secure environment in patient care. Patients can feel more secure if occupational safety is prioritized [22].

Challenges 

Organizational culture refers to the shared beliefs, values, and behaviors within a hospital. A lack of emphasis on patient safety in organizational culture can hinder initiatives that aim to ensure patient safety. It may manifest itself as a lack of commitment, inadequate support, or insufficient prioritization of safety measures by the hospital's leadership and staff. This can result in a higher likelihood of medical errors and adverse events occurring. A weak organizational culture can also discourage staff from reporting incidents or speaking out about potential safety concerns further compromising patient safety [23].

Effective communication is crucial in healthcare settings. Inadequate or ineffective communication between healthcare professionals can lead to misunderstandings, errors in medical decisions, and improper patient care. Clear and concise communication is necessary to ensure the accurate transfer of information between team members. It is essential for healthcare professionals to communicate efficiently not only within their team but also with patients and their families [24]. Leadership’s approach and commitment to patient safety significantly impact the hospital culture. If leaders do not prioritize or actively foster a culture of safety, it can negatively affect staff engagement and commitment to patient safety practices. Strong and supportive leadership is crucial for implementing and maintaining a culture that prioritizes patient safety [25]. Individual factors such as attitudes, beliefs, and behaviors can hinder the adoption of patient safety practices. Resistance to change, lack of awareness, or different perceptions among staff about the importance of safety measures can impede the establishment of a unified patient safety culture [26].

Insufficient resources, including staffing, technology, and training, pose significant challenges to patient safety. Limited resources might hinder the hospital's ability to implement robust patient safety protocols and invest in the necessary tools or personnel to improve safety measures. As a result, patient safety incidents can occur more frequently, potentially leading to harm or even death. Inadequate staffing levels can lead to overwhelmed healthcare professionals, increasing the risk of compromising quality of care. Insufficient technology and training can also hinder the timely identification and prevention of potential safety hazards, further exacerbating the challenges facing healthcare organizations to ensure patient safety [27]. Staff resistance to adopting new patient safety protocols or practices can hinder progress. Change management is crucial in implementing new safety initiatives. Resistance may arise from various factors such as fear of the unknown, perceived inconvenience, or lack of understanding of the benefits of new safety measures [28].

The high workloads and time constraints faced by healthcare professionals can compromise their ability to fully adhere to patient safety protocols. The demand for efficiency and time management in healthcare care can sometimes conflict with the necessary care and attention necessary to ensure patient safety. This can lead to potential errors or oversights in the follow-up of safety protocols, which put patients at risk. Furthermore, the pressure to meet deadlines and quotas can create a stressful work environment, which can further impact the mental and physical well-being of healthcare professionals, potentially affecting their performance and decision-making abilities [29]. Challenges in patient safety culture are as follows (Table 1).

**Table 1:** Challenges in patient safety culture

Challenges	Description
Organizational culture	Shared beliefs, values, and behaviors that affect patient safety initiatives. Lack of emphasis hinders safety measures and discourages incident reporting [23].
Communication	Vital to accurate information transfer among healthcare professionals, patients, and their families [24].
Leadership commitment	Influence of leadership on staff engagement and commitment to patient safety practices [25].
Individual factors	Attitudes, beliefs, and behaviors hinder the adoption of safety practices, leading to resistance to change [26].
Insufficient resources	Staffing, technology, and training deficits impact safety protocols, potentially leading to more incidents [27].
Resistance to change	Staff resistance to adopting new safety measures due to various factors [28].
Time pressures	Workloads and time constraints that compromise adherence to safety protocols, affecting patient safety [29].

Importance of patient safety 

Healthcare organizations undergo evaluations based on patient experience and safety to ensure that they provide compassionate, effective, and safe care. According to national rules and regulations, the General Medical Council, quality indicators, and local monitoring measures are used [30]. Patient safety is intended to reduce the frequency of negative conclusions, medical mistakes, and patient injuries that can be avoided. Healthcare workers can reduce the chances of difficulties, injuries, and even deaths by implementing rules and safety policies in practice. Hospital systems are recognized as the most important settings for reducing medical errors and promoting the quality of patient care [31]. Better patient outcomes are the result of safe care. Patients are more likely to have excellent medical results, better recovery rates, fewer problems, and shorter hospital stays when they receive high-quality and safe care. Patient feedback can have an impact on the overall quality improvement strategy [32].

Patient safety is important to maintain trust between patients and healthcare providers. Patients are more likely to have faith in the healthcare system when they feel comfortable and secure during their healthcare experience. Competency is classified in the medical and nursing literature according to knowledge, skills, and attitudes. Knowledge refers to the ability of a healthcare professional to identify and understand potential patient safety characteristics. Improving healthcare safety is a global priority and the problem is approaching epidemic proportions [33,34]. Patient safety measures can help reduce healthcare costs associated with avoided mistakes and adverse outcomes by investing in patient safety. Medical errors tend to result in additional treatments, long hospital stays, and legal expenses, leading to higher healthcare costs compared to standard medical care [35].

The potential impact of changes in the system of payments on health outcomes is uncertain due to very little certainty evidence. Insufficient data was collected to examine the impact of the design of certain aspects of the payment system such as the size of the incentive and the type of performance measures. Furthermore, given limited and very low certainty evidence, it is unclear whether changing payment structures without providing additional money to researchers will result in a similar effect [36]. Patient safety plans foster a culture of continuous improvement within healthcare organizations. We included randomized trials that investigated a program to improve outpatient treatment for patients. The programs were required to study at least one system or provider-targeted strategy alone or in combination with a patient-targeted strategy [37]. The need to broaden the scope of participation of patients in primary care improvement remains [38]. Hospitals demonstrate that their organizations have invested in patient improvement measures [39]. The legal and regulatory standards for patient safety apply to healthcare organizations. Changing health expectations, increasing public expectations, and new innovative health goals challenge health systems to deliver better health outcomes and greater social value [40].

In the regulation of healthcare care, the problem of patient and family involvement becomes more and more important. The nature of user participation in the regulation may vary. For therapeutic measures, those who practice depend on the precision and quality of the information provided by patients. Consequently, healthcare care providers feel obligated to keep enormous amounts of sensitive personal information about their patients. Similarly, patients believe that personal information provided for medical reasons will remain private. A database that provides services like data download and access is referred to as a data provider. When sensitive and personal data are involved, the data provider becomes a data controller under the data protection directive [41]. Table 2 shows aspects of patient safety.

**Table 2:** Aspects of patient safety

Aspect	Description
Minimizing harm	Implementing rules and safety policies to reduce medical errors and injuries, especially within hospital systems [31].
Improved health outcomes	Safe care leads to better medical results, recovery rates, fewer complications, and shorter hospital stays. Patient feedback helps improve quality [32].
Building trust and confidence	Vital for patient-provider trust. Competency in healthcare personnel enhances safety awareness and global healthcare prioritization [33,34].
Cost reduction	Patient safety measures minimize healthcare costs associated with errors and adverse outcomes compared to standard care [35,36].
Continuous improvement	Safety plans cultivate a culture of continuous improvement. Trials focus on improving outpatient care and patient involvement in primary care [37-39].
Legal and regulatory requirements	Healthcare organizations adhere to legal standards; evolving health expectations emphasize patient and family participation and data protection [40,41].

Employee education and training on patient safety

Employee education and training in patient safety is an important component of healthcare systems that contributes to the development of a patient safety culture. It empowers healthcare workers with the knowledge, skills, and attitudes needed to detect and reduce potential risks, prevent errors, and ensure the well-being of patients. To reduce the anxiety of reporting sentinel incidents and increase overall patient safety, a culture change is required by advising a blame-free culture and building collaboration handovers and communication openness [42]. A combination of different elements has culminated in improved health economics and more efficient utilization of healthcare services as well as improved quality, relationships, and customer and staff experiences for patients [43]. Education and training on patient safety are described in Table 3.

**Table 3:** Education and training

Education and training	Description
Importance of training	Training programs are important in developing patient safety and awareness of reduction awareness. Patient participation is essential to national and international patient safety programs [44].
Interprofessional training	Professionals from many different fields, such as physicians, nurses, chemists, technicians, and support workers, should participate in patient safety training. The patient safety culture is an accumulation of shared values and policies shared by all professionals in the professionals of organization's professionals [45].
Continuous education	Patient safety training is an ongoing process, not a one-time event. Healthcare worker and patient safety is a connected problem, especially in hospitals. A thorough understanding of the safety culture includes both the workplace and patient safety cultures [46].
Evaluation and feedback	Regular evaluation of staff education initiatives and participant feedback must be performed to measure the effectiveness of training activities. The value of safety culture has been associated with multiple critical clinical outcomes and patient satisfaction [47].

## Conclusions

Patient safety is the center of attention in the healthcare landscape requiring meticulous attention and strategic interventions. Through rigorous evaluation through randomized trials, programs have emerged as crucial in enhancing outpatient treatment and fostering safer patient environments. Training programs play an essential role in cultivating safe environments and minimizing misunderstandings. The dynamic culture of patient safety within hospitals acts as a catalyst, substantially improving quality, performance, and productivity in healthcare. These initiatives enable hospitals to drastically reduce medical errors, improve patient outcomes, and streamline operations. To promote patient safety, staff education and training, effective communication, medication safety, infection control, and emergency preparedness must be addressed to prevent future incidents. We need to ensure collaborative understanding and closed-loop communication between healthcare teams. Despite challenges such as weak organizational culture, practical communication, and resource limitations, the importance of patient safety remains paramount, aligned with improved health outcomes, trust, cost reduction, continuous improvement, and compliance with regulations. Education and training of healthcare professionals emerge as the cornerstones of fortifying systems, equipping them with the skills and mindset to ensure patient safety through collaboration and transparent communication.
